# Basalt Fiber-Based Flame Retardant Epoxy Composites: Preparation, Thermal Properties, and Flame Retardancy

**DOI:** 10.3390/ma14040902

**Published:** 2021-02-14

**Authors:** Yu Guo, Meihui Zhou, Guang-Zhong Yin, Ehsan Kalali, Na Wang, De-Yi Wang

**Affiliations:** 1 Sino-Spanish Advanced Materials Institute, Shenyang University of Chemical Technology, Shenyang 110142, China; luckyluckyflora@163.com (Y.G.); iamzhoumeihui@126.com (M.Z.); 2 Materials Science and Engineering Area, Universidad Rey Juan Carlos, Calle Tulipan, s/n, 28933 Móstoles, Spain; 3 IMDEA Materials Institute, C/Eric Kandel, 2, 28906 Getafe, Madrid, Spain; guangzhong.yin@imdea.org (G.-Z.Y.); ehsan.kalali@imdea.org (E.K.)

**Keywords:** epoxy resin (EP), basalt fiber (BF), flammability, composites

## Abstract

We aimed to study the impact of surface modification of basalt fiber (BF) on the mechanical properties of basalt fiber-based epoxy composites. Four different types of pretreatment approaches to BF were used; then a silane coupling agent (KH550) was applied to further modify the pretreated BF, prior to the preparation of epoxy resin (EP)/BF composites. The combination of acetone (pre-treatment) and KH550 (formal surface treatment) for basalt fiber (BT-AT) imparted the EP/BF composite with the best performance in both tensile and impact strengths. Subsequently, such modified BF was introduced into the flame-retardant epoxy composites (EP/AP750) to prepare basalt fiber reinforced flame-retardant epoxy composite (EP/AP750/BF-AT). The fire behaviors of the composites were evaluated by vertical burning test (UL-94), limiting oxygen index (LOI) test and cone calorimetry. In comparison to the flame-retardant properties of EP/AP750, the incorporation of BF-AT slightly reduced LOI value from 26.3% to 25.1%, maintained the good performance in vertical burning test, but increased the peak of the heat release rate. Besides, the thermal properties and mechanical properties of the composites were investigated by thermogravimetric analysis (TGA), universal tensile test, impact test and dynamic mechanical analysis (DMA).

## 1. Introduction

As a thermosetting polymer, epoxy resin (EP) became a preferred matrix for electric insulation, coatings, laminates, and composites due to its outstanding advantages such as high thermal conductivity, low shrinkage, mechanical stabilities, easy handling, chemical resistance, and excellent physical properties. Nevertheless, the intrinsic brittleness and high flammability still limit its application for several advanced areas. In order to tackle this issue, some approaches have been conducted to improve these drawbacks, such as incorporation of nanocarbon materials [1], layered double hydroxides (LDHs) [2,3], metal–organic frameworks (MOFs) [4,5], nanoclay [6,7], and phosphorus-containing flame retardants [8,9]. Concerning the environmental friendliness and high-efficiency, intumescent fire retardants (IFRs) have been long-term developed and attracted numerous attentions to polymers [10,11,12]. Exolit AP750 (AP750) as a high efficiency intumescent flame retardant because of its machining stability, smoke suppressibility and non-toxic, is used in the following study. 

Basalt fibers (BFs) are inorganic materials with high modulus, good strength, rectified strain to failure, excellent temperature resistance, superb stability, great chemical resistance, and they are easy to process, non-toxic, natural-based, eco-friendly, and cost-effective [13,14,15,16]. BF might be a better reinforced fiber as compared to glass fiber (GF), even carbon fiber (CF) in some conditions. For example, the basalt fiber provided better resistance than the GF at accelerated weathering test, and kept much better performance on the strength than both of CF and GF after exposure at 600 °C for 2 h [16]. However, due to the poor adhesive property of BF with EP, the application of basalt fiber-epoxy composite is very limited. The introduction of modified basalt fiber has been considered as one of the most effective methods to improve the performance of EP composites [17,18,19]. Nevertheless, in comparison to the wide studies in the state of the art related to the GF, CF and/or natural fibers, the investigation on BF and its based epoxy composites is still very limited in both of fundamental and applied aspects. For example, the relationship between varied surface pretreatment of BF and their mechanical performance in the epoxy is still not well studied. In this context, the main objective of this paper is to investigate the relationship between the surface modifications of BF and properties of BF reinforced epoxy composites. 

In this study, firstly, four surface pretreatment approaches to pre-modify the BF, then introduced the BF modified by γ-aminopropyl triethoxysilane (KH550) into the EP to prepare basalt fiber reinforced epoxy composites. Secondly, the modified BF via optimized pretreatment approach and KH550 was added to flame retardant epoxy (EP/AP750) to prepare basalt fiber reinforced EP/AP750, named EP/AP750/BF-AT. Then, the mechanical, thermal and fire properties of these composites were investigated systemically. The main novelty of this study is to clarify the relationship between surface modifications of basalt fiber and the mechanical properties of basalt fiber-based epoxy composites.

## 2. Materials and Methods

### 2.1. Materials

In this work, short basalt fiber (length: 3 mm) was cutting from continuous one and supplied by Anjie Composite Materials Co. Ltd. (Haining, China). Acetone, glacial acetic acid and sodium hydroxide (NaOH) were purchased from Fuyu Fine Chemical Industry Co. Ltd. (Tianjin, China). γ-aminopropyl triethoxysilane (KH550) was provided by TCI Chemicals Company (Shanghai, China). Exolit AP750 as flame retardant used in this study was purchased from Clariant AG (Frankfurt, Germany). Epoxy resin (trade name E-44) as the matrix resin was offered by Nantong Xingchen Synthetic Material Co. Ltd. (Nantong, China). The curing agent 4,4’-Diaminodiphenyl sulfone (DDS) was supplied from Sinopharm Chemical Reagent Co. Ltd. (Shanghai, China).

### 2.2. Preparation of Modified Basalt Fibers

Before the basalt fiber was modified formally by silane, pretreatment to the fiber was carried out in this study in order to clean the surface. By referring to the previous studies [20,21,22,23], four different pretreatment approaches were used to basalt fibers:

(1) 10 g basalt fibers were immersed in 200 mL acetone solution ultrasonic cleaning at 50 °C for 3 h, then the fibers were soaked in acetone for 21 h and dried for 48 h at 60 °C after the filtration.
(2) 10 g basalt fibers were immersed in 150 mL glacial acetic acid ultrasonic and cleaned at 50 °C for 3 h using sonication bath. Then, the fibers were soaked in glacial acetic acid for 21 h. The pretreated basalt fiber was rinsed with deionized water to achieve a neutral pH, dried for 24 h at 60 °C after the filtration.
(3) 10 g basalt fibers were added to the mixture of NaOH and deionized water with a mass ratio of 1:20. The mixtures was sonicated at 90 °C for 1 h. The fibers were then diluted and washed with deionized water until the pH = 7. The treated fiber was dried in an oven for 3 h at 60 °C.
(4) 10 g basalt fibers were put into the muffle furnace, which were sealed and heated to 350 °C for 2 h. After that, the pretreated basalt fiber was added to the mixture of deionized water, anhydrous ethanol of analytical purity.

After that, the pretreated basalt fiber was added to the mixture of deionized water, anhydrous ethanol and KH550 with a mass ratio of 30:70:1 for 30 min. The fiber was dried in an oven at 100 °C for 3 h. The modified basalt fibers obtained by four different pretreatment methods were respectively (a) BF-AT meant BF was pretreated by acetone; (b) BF-AAT meant BF was pretreated by glacial acetic acid; (c) BF-SHT meant BF was pretreated by mixture of NaOH and deionized water; (d) BF-MFT meant BF was pretreated by muffle furnace. 

### 2.3. Preparation of Flame Retardant Modified Fiber Epoxy Resin (EP/AP750/BF-AT)

EP, AP750, and BF were mixed according to the formulations in Table 1. The detailed preparation process could be described as following: Firstly, 6.78 g modified basalt fiber was incorporated into 100 g epoxy matrix using a collector type constant temperature heating magnetic agitator for 30 min at 150 °C; Secondly, 13.57 g AP750 was added to the above mixture with 770 rpm for 5min, and then added to 35.68 g DDS for 30 min until DDS totally dissolved. In order to remove the bubbles, the mixture was stewing for 5 min and then immediately poured into the pre-heated silicon rubber molds. The sizes of the molds corresponded to the description of varied tests in 2.4. The curing procedure was set as 160 °C for 1 h, 180 °C for 2 h and 200 °C for 1h. In comparison, the pure EP and EP/AP750 was prepared according to the above procedure described for the composites.

### 2.4. Characterization of Morphology, Structure, and Properties

Surface morphologies of the basalt fibers, before and after the modification were characterized by Hitachi SU8010 model scanning electron microscopy (SEM, Hitachi, Tokyo, Japan), with an acceleration voltage of 5.0 kV. Fourier transform infrared spectra (FTIR) were obtained on Nicolet MNGNA-IR560 (Artisan Technology Group, Austin, TX, USA) with range from 4000 cm^−1^ and 400 cm^−1^ and use attenuated total reflectance method (ATR). A universal tensile testing machine TCS-200 (ISO 37/2017, Gotech, Taiwan, China) was utilized to test the tensile strength with the dumbbell-shaped specimens of 30.0 mm × 5.0 mm × 2.0 mm at 2 mm/min test speed. The impact tests of unnotched specimens with dimensions of 80.0 mm × 10.0 mm × 4.0 mm were carried out (ISO 2932003, Gotech Testing Machines Inc., Taiwan, China). The dynamic mechanical thermal behaviors of epoxy composites with sizes of 40.0 mm × 10.0 mm × 2.0 mm were tested by using a DMA Q800 Dynamic Mechanical Analyzer (New Castle, DE, USA) from 25 to 250 °C at a rate of 3 °C /min with a single cantilever mode and a frequency of 1 Hz. The thermal stability was measured using a thermogravimetric analyzer (TGA) instrument on a STA 449C thermal analyzer (Selb, Germany) at a linear heating rate of 10 °C /min under N_2_ flow. Limiting oxygen index (LOI) values of all the specimens with the sizes of 120.0 mm × 6.5 mm × 3.2 mm were achieved according to ASTM D2863-2013 by LOI tester (FTT, Sussex District, UK). Vertical burning test (UL-94) were carried out by using the standard samples with the dimension of 120.0 mm × 13.0 mm × 3.2 mm according to ASTMD 3801-2010 standard in a burning chamber (FTT, Sussex District, UK), which was an older version of UL-94 standard. The cone calorimeter tests to the samples were carried out on a cone calorimeter (FTT, Sussex District, UK). The squared specimens (100.0 mm × 100.0 mm × 3.0 mm) were wrapped with aluminum foil and irradiated with a heat flux of 50 kW/m^2^, following the ISO 5660-1:2015 standard. For cone calorimeter test, each sample has been tested at least 2 times.

## 3. Results and Discussion

### 3.1. Effect of Fiber Pretreatment on Surface Organics

#### 3.1.1. SEM Analysis of Basalt Fiber

**Figure 1:**
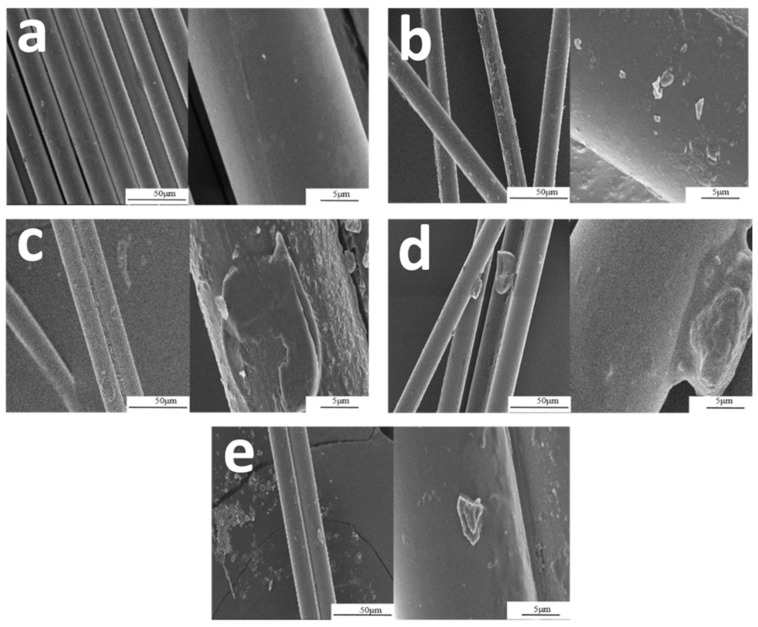
Scanning electron microscopy photographs of outer surface of the basalt fiber after pretreatment: (**a**) BF; (**b**) BF-MFT; (**c**) BF-SHT; (**d**) BF-AT; (**e**) BF-AAT.

In order to understand the compatibility between the epoxy matrix and fibers, the surface morphology and microstructure of BF, BF-MFT, BF-SHT, BF-AT, and BF-AAT were characterized by SEM. As shown in Figure 1a, the samples containing untreated fibers exhibited a relatively smooth surface with few infiltrating agents and impurities. Due to the chemical inertness of basalt fiber to the epoxy, the fiber might be easily detached from the epoxy when it was subjected to dynamic loads. As a result, the interface between the epoxy matrix and the fiber became a defect, which induced a reduction in the mechanical strength compared with the pristine epoxy [24,25]. The morphology of BF-MFT, BF-SHT, BF-AT, and BF-AAT are shown in Figure 1b–e. Figure 1b showed that trivial organic materials adhere to the fiber surface. After high temperature treatment on the basalt fiber, a decrement in the mechanical strength was observed. The altered composition of the fiber increased the electrostatic interactions. The aggregation of basalt fiber was obvious during processing, providing a weak enhancement effect on the epoxy resin [26]. As shown in Figure 1c, the surface of basalt fiber was relatively rough, indicating that the sodium hydroxide had etched the surface of the fiber. Moreover, in Figure 1e, similar phenomenon was found, possibly due to the etched by glacial acetic acid. The good compatibility between the fiber and EP was expected through the AT treatment (Figure 1d), since the modified surface of basalt fibers by the coupling agent seemed better than others did. 

#### 3.1.2. FTIR and TG Analysis of Basalt Fiber

**Figure 2:**
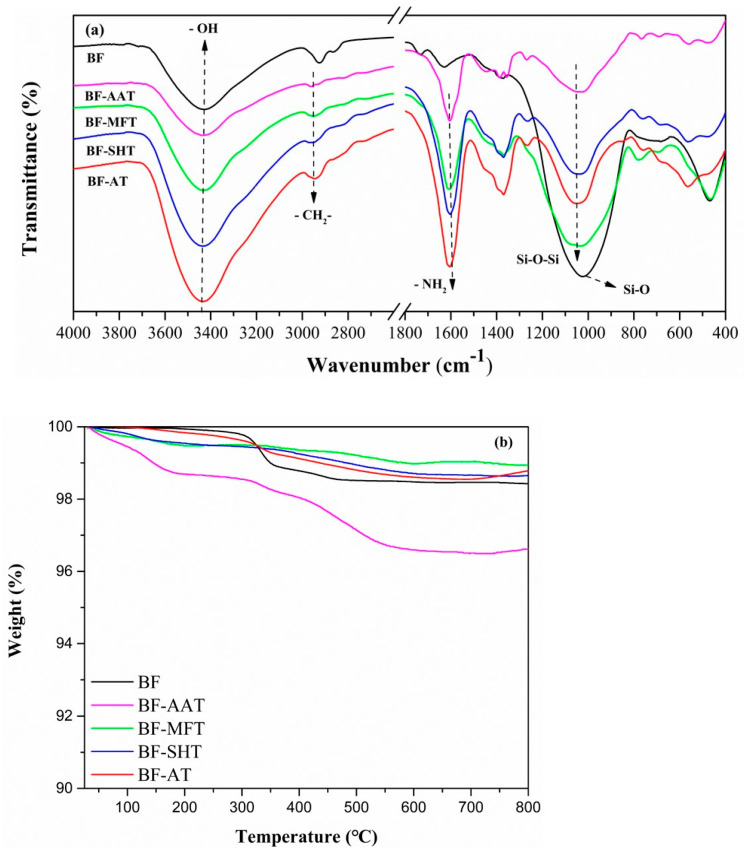
(**a**) FTIR spectra of BF and modified BF; (**b**) thermogravimetric analysis (TGA) of BF and modified BFs.

FT-IR spectroscopy was used to observe the surface of the KH550 modified basalt fibers. As shown in Figure 2a, the peak located at 3438 cm^−1^ was appeared in all samples, which was attributed to stretching vibration of –-OH group from the surface of BF [27]. Silane coupling agent was more likely to react with Si-OH on the BF surface to form Si-O-Si bond. Therefore, increasing the content of Si-OH chemical groups on the surface of BF improved the surface modification effect of KH550 on BF [28]. After acetone pretreatment, the peak area spectrum of the BF-AT was superior to that of other fibers, indicating that the acetone pretreatment had more exposed –OH group on the BF. The absorptions at 2950 cm^−1^ and 1600 cm^−1^ were attributed to the –CH_2_- and –NH_2_ bonds in the silane coupling agent [29]. In the BF-AT spectrum, a feature appeared at 1070 cm^−1^ was Si-O-Si bond in BF, respectively indicating the KH550 was successfully grafted onto the BF surface. 

Figure 2b showed the TGA results of modified basalt fibers under nitrogen atmosphere. As compared to the pure BF, it clearly showed all the modified BFs (BF-AAT, BF-MFT, BF-SHT, BF-AT) owned relatively early weight loss, while most of the modified BFs (BF-MFT, BF-SHT, BF-AT) excepted for the BF-AAT had kept similar or slightly higher residue at high temperature. First of all, the early weight loss to all the modified BFs meant the presence of organic coating, such as KH-550, indicating the organic modification to BF was successful [30]. Moreover, to most of modified BFs such modification did not impact greatly on final amount of residue at high temperature. This meant the quantity of the organic coatings on the fiber is very low. Interestingly, after 330 °C these modified BFs (BF-MFT, BF-SHT, BF-AT) showed even higher thermal stability as compared to that of the pure BF. A possible reason was the formed Si-O-Si network caused by KH-550 on the surface of fiber reacted as protection layer to the fiber. Regarding the exceptional case, BF-AAT, it might be due to relatively high amount of organic compound, such as glacial acetic acid, contained during the fiber modification. This phenomenon was similar to the modification of magnesium hydroxide [31].

### 3.2. Effect of Modified Basalt Fiber on the Mechanical Properties of Epoxy Composites

#### 3.2.1. Tensile and Impact Properties

**Figure 3:**
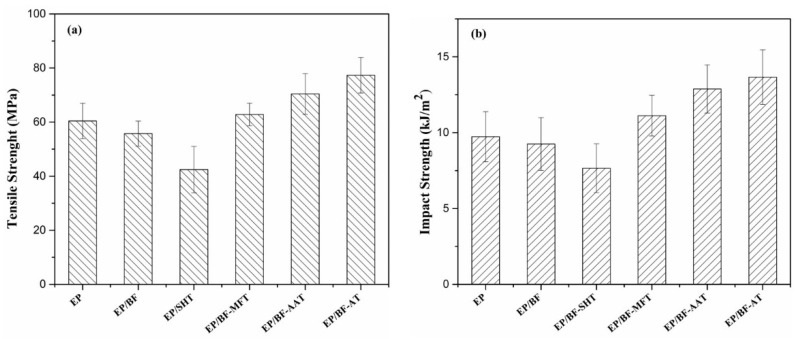
(**a**) Tensile strength, (**b**) unnotched impact strength behavior of epoxy-based composites.

The variation of tensile and impact strength of epoxy-based composites with basalt fiber were associated with various pretreatment approaches of specimens. Figure 3 showed that the addition of pure basalt fiber and the one etched via NaOH (SHT-fiber) reduced the tensile and impact strengths of epoxy matrix compared with the rest composites. As aforementioned in SEM analysis, the rough surface was observed for SHT-fiber, which resulted in the poor tensile and impact strength. Also, in the case of EP/BF composites, as compared to the pure epoxy, it showed lower strength in both of tensile and impact tests due to a poor interaction between the epoxy resin and basalt fiber. Compared with sample EP/SHT, the rest modified BF based epoxy composites showed better performance in the two tests, in particular to the EP/BF-AT. Clearly, the difference in the mechanical properties amongst these samples was corresponding to the varied pretreatment of the surface prior to the modification by using KH550. Since EP/BF-AT showed the best properties in both tensile and impact tests, which meant using acetone as pretreatment agent was a good option aiming at developing high performance basalt fiber. This pretreatment may efficiently assist the purification of the surface of basalt fiber without damaging its intrinsic properties; it would provide a good condition on the surface of fiber for further modification by silane. In this context, the well-modified basalt fiber led to good interaction between the basalt fiber and epoxy resin. These results indicated that the pretreatment for the surface of basalt fiber prior to the formal modification via silane are very important. Notably, it is a crucial step to develop high performance basalt fiber-based composites. 

#### 3.2.2. Dynamic Mechanical Analysis (DMA)

**Figure 4:**
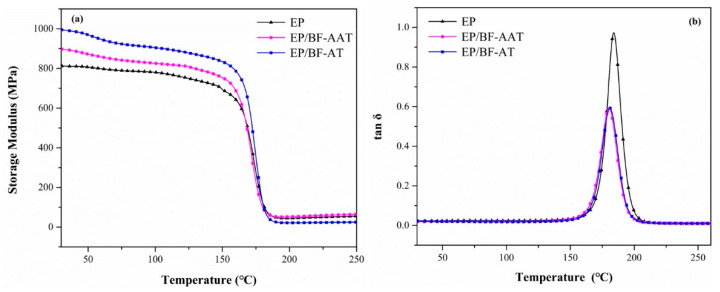
Dynamic mechanical thermal behavior of epoxy-based hybrid composites: (**a**) Plots of storage modulus versus temperature; (**b**) plots of tan delta versus temperature.

DMA was used to study the thermomechanical properties of the cured epoxy networks, it allowed to evaluate the following parameters: Storage modulus, G’, loss modulus, G”, and mechanical loss factor (tan δ) [32]. The storage modulus and tan delta values of the EP/BF-AAT and EP/BF-AT were shown in Figure 4. The storage modulus of EP/BF-AT demonstrated considerably higher value than that of EP/BF-AAT below 160 °C. The remarkably enhanced storage modulus approved that EP/BF-AT owned relatively better performance than that of EP/BF-AAT. Although the mechanism behind of this phenomenon is not clear yet and needs to be further study in the future, it clearly approves that the pretreatment for basalt fiber prior to the formal surface modification is very important. This finding may be different as compared to other fibers, such as glass fiber, carbon fiber, etc. Possibly, based on the pretreatment via acetone the surface of basalt fiber is well purified and easy to be further modified by KH550, which led to a good interaction between the basalt fiber and epoxy resin. In contrast, EP/BF-AAT showed a lower storage modulus, indicating a relatively poor interaction between the epoxy matrix and basalt fibers based on the pretreatment of surface by glacial acetic acid to the fiber. Furthermore, glass transition temperature (T_g_) was determined by the peak of tan θ curves. It was noted that the T_g_ values of EP/BF-AT (180.1 °C) and EP/BF-AAT (180.4 °C) were obviously lower than that of pure EP (191.3 °C). It might be related to the change of crosslinking density due to incorporation of basalt fiber modified by KH550, since a reduced crosslinking density can be responsible to a decreased T_g_ [33]. However, the real reason has to be further confirmed by a comprehensive study in the future, such as analyzing all the epoxy-based composites in this study by both of DMA and DSC. 

### 3.3. Thermal Degradation Behavior of Flame-Retardant Epoxy Resin

**Figure 5:**
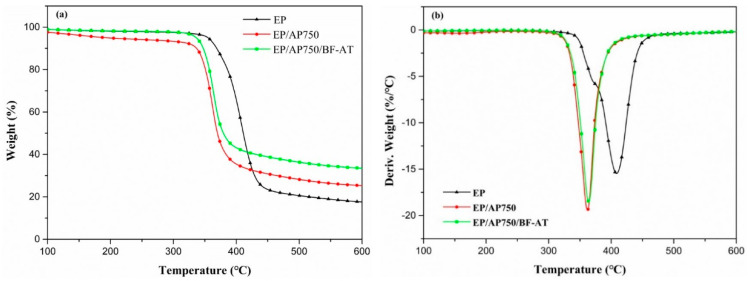
(**a**) TG and (**b**) DTG curves of pure EP and flame-retardant epoxy under N_2_ at heating rate of 10 °C /min.

Thermogravimetric analysis (TGA) had been often used to investigate the thermal decomposition behaviors of polymer composites. Herein, the thermal degradation behaviors of EP, EP/AP750, EP/AP750/BF-AT were investigated by TG/DTG under nitrogen condition, and the curves were shown in Figure 5. The corresponding data were summarized in Table 2 including the initial decomposition temperature (T_5%_), the temperature at 50 wt% mass loss (T_50%_), the temperature at maximum weight loss rate (T_max_), the max degradation rate (R_max_) and the char residues at 600 °C. For EP, it showed a single thermal decomposition stage, starting at 360°C, and almost immediately reached its T_max_ at 408 °C with a R_max_ of 22 wt%/°C and a char residue of 17.3% at 600 °C. There was visible increase in T_50%_ and char yield of both EP/AP750 and EP/AP750/BF-AT. 

As compared with EP, EP/AP750 showed a lower T_5%_, T_max_ and a higher char residue (25.3%) at 600 °C. The earlier initial thermal decomposition of EP/AP750 was mainly caused by the early decomposition of AP750, which produced polyphosphoric acid and led to accelerate the decomposition of EP matrix [34]. At elevated temperature, polyphosphoric acid like compounds could further produce branched and cross-linking reaction and catalyze the carbonization of EP molecules to form more stable protective char layer. Furthermore, the char yield increased from 25.3% for EP/AP750 to 33.5% for EP/AP750/BF-AT. However, the amount of basalt fiber (around 6%) as inorganic filler in the epoxy composites had to be taken into account, indicating that there was almost no synergistic effect between AP-750 and basalt fiber in the char formation. This is reasonable since the basalt fiber is non-active and quite thermal-stable (less than 2% weight loss at 700 °C).

### 3.4. Effect of Modified Fiber on Combustion Performance of Epoxy Resin

#### 3.4.1. Flammability

Investigation of the flammability of the pristine EP and EP-based composites was performed by limiting oxygen index (LOI) and UL-94 vertical burning test. As listed in Table 3, the LOI value of the pristine EP was 23.2% and there was no classification in the UL-94 vertical burning test. After adding AP750 into epoxy, as expected the EP/AP750 showed improved LOI value (26.3%) and short burn times and without dripping during the test. However, as incorporating the BF-AT, the EP/AP750/BF-AT exhibited a slight reduction in the LOI value of 25.1%, while the sample kept the similar phenomenon in the UL-94 test as EP/AP750. These results meant the introduction of BF-AT into EP/AP750 only had minor impact in the LOI test. This conclusion may be important to developing some compromised solution to flame retardant epoxy composite if both of vertical burning behaviors and mechanical reinforcement are the main concerns to the composites. Moreover, the slightly reduced performance of EP/AP750/BF-AT during the LOI test could be due to the “candlewick effect” caused by basalt fibers in the combustion process, which is similar to glass fiber reinforced polymer composites [35].

#### 3.4.2. Fire behaviors

**Figure 6:**
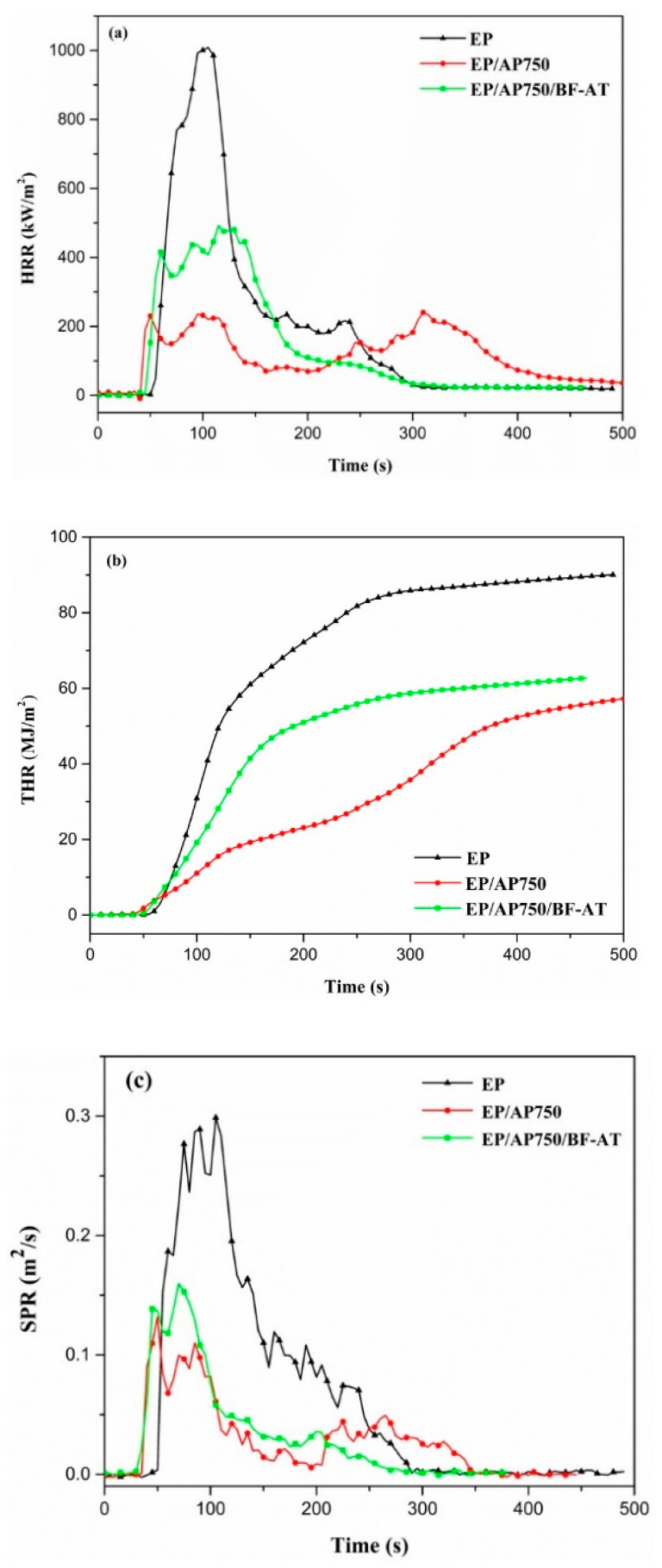
Cone calorimeter results of EP, EP/AP750 and EP/AP750/BF-AT in (**a**) heat release rate (HRR), (**b**) total heat release (THR), and (**c**) smoke produce rate (SPR).

Cone calorimeter test (CCT) was a fire simulation testing based on the oxygen consumption principle, which conducted to investigate sufficiently the combustion performance of EP, EP/AP750 and EP/AP750/BF-AT composites. The curves of heat release rate (HRR), total heat release (THR), smoke produce rate (SPR) and total smoke production (TSP) were all shown in Figure 6. The main characteristic parameters, such as the time to ignition (TTI), the peak of HRR (pHRR), average effective heats of combustion (EHC), the peak of SPR (pSPR), char yields, fire performance index (FPI), maximum average heat release rate (MARHE), and fire growth rate index (FIGRA) were summarized in Table 4.

Figure 6a,b showed the HRR and THR curves of neat EP and flame-retardant EP composites. The HRR curve of neat EP presented a sharp peak during a short time range (from 80 to 150 s) and its peak HRR (pHRR) reached to a value of 950 kW/m^2^. It suggested that the process of heat release of neat EP was drastic and rapid. Meanwhile, EP/AP750 had a pHRR of 273 kW/m^2^, decreased by 71.3% than that of pure EP. In comparison, the introduction of BF-AT into EP/AP750 kept similar time to ignition (TTI), while the value of pHRR increased to 458 kW/m^2^. However, after 210 s the HRR curves of EP/AP750 and EP/AP750/BF-AT showed interestingly difference; EP/750/BF-AT exhibited better behavior in the final stage of the combustion which was mainly due to the certain amount of basal fiber contained in the residue as inorganic phase. 

If we compared the results of MARHE and FIGRA from Table 4 in both of EP/AP750 and EP/AP750/BF-AT. It seemed that the introduction of BF-AT into the EP/AP750 worsened the performance in the combustion. 

Another hazard associated with the combustion of EP is the heavy smoke produced during combustion, which cause people death by suffocation or inhalation of the toxic gases. Smoke production were urgent issues for flame-retarded polymers, since they were the fatal factors in a fire. Smoke production release (SPR) curves were presented in Figure 6c and the detailed values of pSPR were shown in Table 4. It was shown that the trend of SPR curves of EP composites was similar to those in HRR results. In comparison, both of EP/AP750 and EP/AP750/BF-AT showed much lower peak SPR as compared to that of pure EP. Nevertheless, the incorporation of BF-AT into EP/AP750 did not show the positive effect on the smoke suppression. This conclusion needs to be taken into account in the formulation design of flame retardant epoxy composites for some potential industrial application.

**Figure 7:**
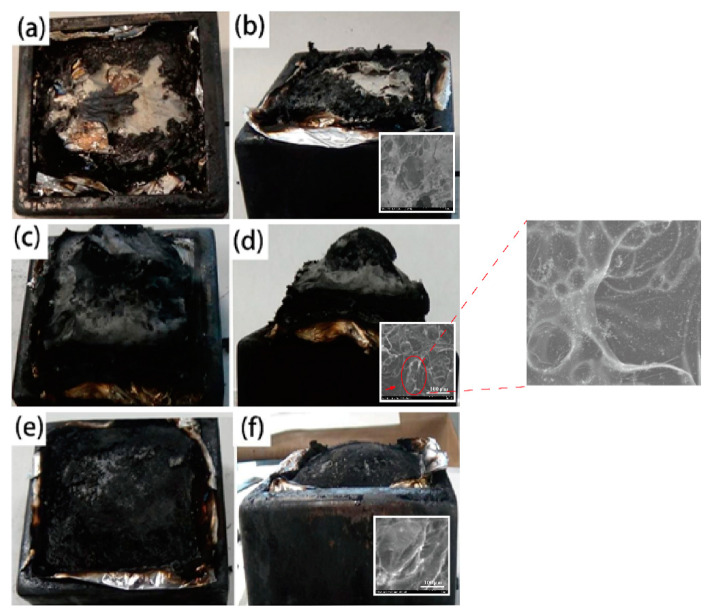
The digital photographs of residue after cone calorimeter test: (**a**,**b**) EP; (**c**,**d**) EP/AP750; (**e**,**f**) EP/AP750/BF-AT.

In addition, the digital images of the residues of EP, EP/AP750, and EP/AP750/BF-AT samples after cone calorimeter testing (CCT) were shown in Figure 7. Clearly, the EP had less residue with plenty of holes and/or cracks on the surface of the residue. The residue from EP/AP750 was typical but high intumescent char layer due to the presence of AP-750. As expected, such char layer formed during the combustion of EP/AP750 as physical barrier ensured the improvement of flame retardancy. In comparison to EP/AP750, EP/AP750/BF-AT showed different char forming behaviors. In details, the intumescent behaviors of EP/AP750/BF-AT during the combustion test was greatly suppressed due to the barrier effect of basalt fiber in the carbonaceous, as shown in Figure 7e. This should be the main reason that led to the worse performance after introduction of BF-AT into EP/AP750 in the cone calorimeter test.

### 3.5. Mechanical Properties of EP/AP750 and EP/AP750/BF-AT

**Figure 8:**
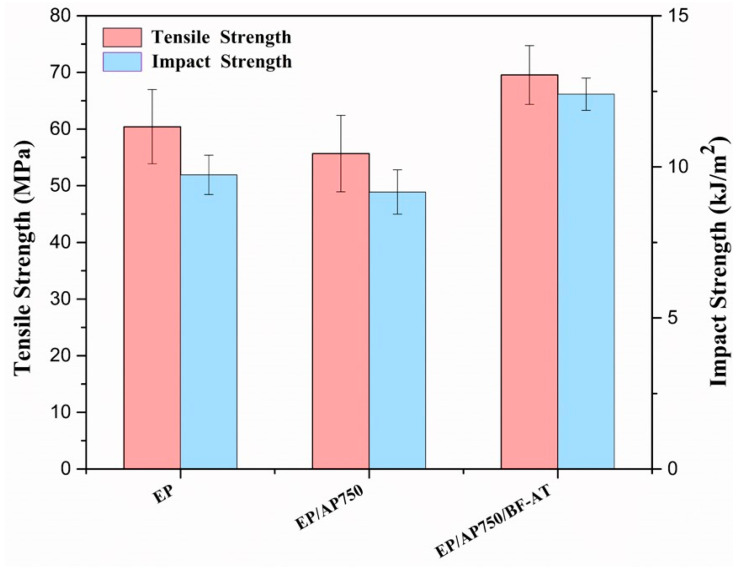
Tensile and impact results of EP, EP/AP750, and EP/AP750/BF-AT.

Mechanical properties of epoxy and flame-retarded epoxy composites were analyzed by tensile and impact tests and the results were plotted in Figure 8. The tensile and impact properties of the EP/AP750 decreased as compared to EP, due to the introduction of AP750. It may be related to the poor compatibility between the AP750 and epoxy resin. Therefore, how to make proper surface modification for flame retardant is also an important topic, in order to improve the interface between two phases. Compared with sample EP, the addition of modified basalt fiber (BF-AT) into EP/AP750 obviously improved both of the tensile and impact strengths by 15.2% and 27.8%, respectively. It meant two steps surface modification, pretreatment by acetone and further modification by KH550, to basalt fiber was an efficient approach to improve the interaction between the basalt fiber and resin. 

## 4. Conclusions

Four different types of pretreatment approaches to BF were used prior to the surface modification by KH550. The modified BFs were named BF-AT, BF-AAT, BF-SHT, and BF-MFT, respectively. Subsequently, these modified BFs based epoxy composites (EP/BF-AT, EP/BF-AAT, EP/BF-SHT, and EP/BF-MFT) were prepared. Clearly, the combination of acetone (pre-treatment) and KH550 (formal surface treatment) for basalt fiber (BT-AT) imparted the EP/BF composite best performance in both of tensile and impact strengths. These results meant an optimized pretreatment of surface to BF prior to the formal modification by silane is very crucial. In comparison to the flame retardant properties of EP/AP750, the incorporation of BF-AT slightly reduced LOI value from 26.3% to 25.1%, maintained the good performance in vertical burning test, but increased the peak of the heat release rate due to the barrier effect of BF in the carbonaceous which limited the intumescent behaviors of AP750 during the combustion. The reinforcement caused by the BF-AT to the EP/AP750 was approved in both of tensile and impact tests, but at the expense of an increased heat release in the cone calorimeter test. In order to compensate for the loss of flame retardancy, in the next study either more flame retardant loading may be needed or explore different flame retardants to basalt fiber reinforced epoxy. 

## Figures and Tables

**Table 1:** Formulations of epoxy resin (EP)/basalt fiber (BF)-based composites.

Sample	EP/phr	AP750/phr	BF/phr	BF-AT/phr	BF-AAT/phr	BF-SHT/phr	BF-MFT/phr
EP/BF	100	/	6.78	/	/	/	/
EP/BF-AT	100	/	/	6.78	/	/	/
EP/BF-AAT	100	/	/	/	6.78	/	/
EP/BF-SHT	100	/	/	/	/	6.78	/
EP/BF-MFT	100	/	/	/	/	/	6.78
EP/AP750	100	13.57	/	/	/	/	/
EP/AP750/BF-AT	100	13.57	/	6.78	/	/	/

**Table 2:** Data obtained from TG and DTG curves of EP, EP/AP750, and EP/AP750/BF-AT.

Sample	T_5%_ (°C)	T_50%_ (°C)	T_max_ (°C)	R_max_ (%/°C)	Residue Yield at 600 °C (%)
EP	360	409	408	22	17.3
EP/AP750	338	376	368	19	25.3
EP/AP750/BF-AT	335	378	362	18	33.5

**Table 3:** Limiting oxygen index (LOI) and UL-94 results of flame retardant modified fiber epoxy resin.

Sample	LOI (%)	Burn Time t1/t2 ^a^ (s)	Dripping
Pure EP	23.2 ± 0.2	>30/- ^b^	Yes
EP/AP750	26.3 ± 0.2	1/2	No
EP/AP750/BF-AT	25.1 ± 0.2	1/3	No

^a^ t1/t2: Average flame time after the first/second time ignition; ^b^: No second time ignition applied.

**Table 4:** Cone calorimeter test (CCT) data of EP and EP composites.

Sample	TTI (s)	pHRR (kW/m^2^)	EHC (MJ/kg)	THR (MJ/m^2^)	pSPR (m^2^/s)	Char Yield (wt.%)	FPI (m^2^·s/ kW)	MARHE (kW/m^2^)	FIGRA (kW/(m^2^·s))
EP	46 ± 4	950 ± 21	22 ± 0	83 ± 3	0.285 ± 0.001	8.2 ± 0.6	4.8 ± 0.3	406 ± 13	10.3
EP/AP750	40 ± 6	273 ± 12	20 ± 2	48 ± 5	0.099 ± 0.004	32.8 ± 0.3	14.7 ± 0.8	149 ± 21	1.7
EP/AP750/BF-AT	41 ± 1	458 ± 15	19 ± 1	51 ± 4	0.159 ± 0.002	29.5 ± 0.2	9.3 ± 0.4	240 ± 19	4.7

## Data Availability

Data sharing not applicable.
